# Environmental Durability Enhancement of Natural Fibres Using Plastination: A Feasibility Investigation on Bamboo

**DOI:** 10.3390/molecules25030474

**Published:** 2020-01-22

**Authors:** Daanvir K. Dhir, Armin Rashidi, Grant Bogyo, Ron Ryde, Sepideh Pakpour, Abbas S. Milani

**Affiliations:** 1 School of Engineering, The University of British Columbia, Kelowna, BC V1V 1V7, Canada; ddhir@alumni.ubc.ca (D.K.D.); armin.rashidi@ubc.ca (A.R.);; 2 Ryde Holdings Ltd., Penticton, BC V2C 2R4, Canada; grant_bogyo@telus.net (G.B.); rryde@icloud.com (R.R.)

**Keywords:** plastination, bamboo natural fibre composites, environmental durability

## Abstract

Natural fibers are gaining wide attention due to their much lower carbon footprint and economic factors compared to synthetic fibers. The moisture affinity of these lignocellulosic fibres, however, is still one of the main challenges when using them, e.g., for outdoor applications, leading to fast degradation rates. Plastination is a technique originally used for the preservation of human and animal body organs for many years, by replacing the water and fat present in the tissues with a polymer. This article investigates the feasibility of adapting such plastination to bamboo natural fibres using the S-10 room-temperature technique in order to hinder their moisture absorption ability. The effect of plastination on the mechanical properties and residual moisture content of the bamboo natural fibre samples was evaluated. Energy dispersive x-ray spectroscopy (EDS) and X-ray micro-computed tomography (Micro-CT) were employed to characterize the chemical composition and 3-dimensional morphology of the plastinated specimens. The results clearly show that, as plastination lessens the hydrophilic tendency of the bamboo fibres, it also decreases the residual moisture content and increases the tensile strength and stiffness of the fibers.

## 1. Introduction

Due to growing concerns for environmental issues such as increasing manufacturing pollution, increasing carbon footprint, and increased awareness about the environmental hazards caused by human-made materials like synthetic fibre composites, great demand, and interest for sustainable natural fibre composites have developed over recent years, particularly owing to their low density, low cost, high stiffness, strength-to-weight ratios, and improved sustainability. Examples of such natural fibres include bamboo, hemp, flax, and wool [1,2,3]. There is a progression in the industry to use natural fibres, including for structural applications, in the form of reinforcing fibres and/or fillers, e.g., within polymer/biopolymer matrices, in the place of synthetic fibres. The global market for natural fiber-based materials is expected to reach $10.89 billion by 2024 [4]. Moreover, the natural fiber composites market grew at an annual compound growth rate of 8.2% between 2015 and 2020 [4].

### 1.1. Processing of Natural Fibres

Existing research efforts recognize various physical and chemical processes to enhance the durability and strength of natural fibres [3]. Sinha and Panigrahi [5] found that plasma-treated fibres demonstrate superior surface hydrophobicity and improved their shear strength. Similarly, research on the treatment of natural fibers has involved heating the fibers to temperatures close to those that can cause their degradation and affect their properties [6]. Huber et al. [7] found that electron radiation can improve the interfacial bond between natural fibres and polypropylene (PP) from 21% to 53% due to the generation of free radicals that promote fibre/matrix cross-linking. Further, the beating of the fibres resulted in a 10% increase in kraft fibre/polypropylene strength due to fiber defibrillation and the corresponding increase in the contact area and mechanical interlocking [8].

A vast bulk of natural fibre processing literature has also focused on the chemical treatment of natural fibres that have shown improved physical strengths and interfacial strength properties [3,6]. Chemically, such treatments often involve the use of alkali, acetyl, silane, benzyl, acrylic, permanganate, peroxide, isocyanate, titanate, zirconate, and acrylonitrile treatments, along with a maleic anhydride graft interfacial agent [9]. The alkaline treatment removes fibrous constituents such as lignin, hemicellulose, wax, and pectin, thereby exposing the cellulose and increasing the roughness/surface area, thus improving interfacial bonding [10]. However, much of the research involving the processing/pre-treatment of natural fibres has been carried out to enhance their mechanical and interfacial properties when used in a composite. These techniques, however, do not address the problem of the hydrophobic nature of these fibres, which requires adequate attention for their durable long-term usage.

### 1.2. Bamboo Natural Fibres

Among various natural fibres, bamboo is one of the most desirable options due to its low density and high strength properties. Furthermore, bamboo has over 1450 species, and worldwide bamboo production is approximately 30 million tons/year, the highest among all-natural fibres [1,2,3]. Bamboo also has a small harvesting cycle due to its very high growth rate [11]. Given its rapid growth rate and low carbon footprint during the life cycle, it is also referred to as ″CO_2_ better″, i.e., net CO_2_ negative [12]. As an example, due to its superior strength and fast growth rate, the use of bamboo fibers in composites as reinforcement of concrete offers an enhanced technique to increase the dependence of the construction industry on renewable resources [13]. Bamboo fibres can even exceed the strength of, e.g., glass fibres during bending [2]. Despite the aforementioned desirable attributes of such natural fibre, it is still known to be prone to degradation due to the effect of moisture and weathering through a highly heterogamous and porous material microstructure [11].

### 1.3. Plastination

In anatomical sciences, the long-term preservation of animal and human tissues has been carried out for decades, and among the various preservation techniques, ″plastination″ is a well-established method. First applied in 1977 by Dr. Gunther Von Hagens, plastination, also called forced polymerization, has been extensively employed for preserving different bodies and body parts of living organisms [14,15,16]. In essence, plastination replaces the water and lipids in the tissue with curable polymers. It involves preserving perishable biological specimens using a series of procedures that replaces tissue water and part of tissue fat with curable polymers, mostly silicone, and epoxy that hinder the decay of body tissue.

Silicone plastination (S-10 technique) is one of the simplest and most versatile types of plastination involving silicone polymers as impregnation mixtures and hardeners. Among different silicone polymers used, S-10 (polydimethylsiloxane) is the most popular and widely used polymer and results in more natural-looking, opaque specimens [17,18,19]. The S-10 technique is comprised of four plastination steps, namely fixation, dehydration, forced impregnation, and curing. In this technique, S-3 (dibutyltindilaurate) and S-6 (tetraethoxysilane) are used as a catalytic agent and hardener, respectively. Besides the wide range of applications of plastination techniques among human, animal and yeast tissues, there is no report on whether plastination could be possibly applied to preserve natural fibres as a way to enhance the strength and durability of these fibres and their composites like bamboo fibre reinforced plastic.

### 1.4. Objective and Organization of Present Work

A major drawback of using natural fibres is their durability. Lignocellulosic fibers tend to have hydrophilic groups attached, due to which they absorb a lot of moisture. This moisture weakens the cellulose structure and the cell wall of these natural fibers, which results in the degradation of their properties. Within the current trend of research reviewed above, the authors believe chemically altering the material composition of these fibres could be one of the solutions to overcome the above environmental performance limitation. In pursuit of that goal, this article provides novel insights on the application of the S-10 plastination technique [14,15,16] on bamboo natural fibres. It includes a detailed procedure developed for the plastination of bamboo, as well as techniques for characterizing the mechanical, physical, and chemical properties of plastinated and virgin bamboo (see Section 3 for methodological aspects of the experiments). Section 2 presents the main findings of the conducted experiments, focusing on two key themes: successfully applying plastination to bamboo and characterizing it, and assessing the environmental durability of the plastinated bamboo. Section 3 outlines the main conclusions of the work and suggests pertinent future work. 

## 2. Results and Discussion

The specific bamboo species that was primarily used for our experiments was *Inversa Bambusoide*, also called ′Yellow Strip Timber′. It is a medium-sized Asian timber bamboo that often has a culm diameter of 0.076 m and a height of around 11–14 m [20]. It establishes quickly, forming a large grove in only four to five years [20]. The two-bamboo species were derived from a partnering supplier (Canada’s Bamboo World, Chilliwack, Canada), in the form of raw bamboo culms and fibre bundles. Single bamboo fibres were technically made up of multiple elementary fibres [21].

The initial attempts for the plastination of bamboo were made using the raw bamboo culms and the fibre bundles using the room temperature S-10 procedure. As the feasibility of the process proved to be successful, some experiments were conducted to optimize the time duration of this natural fibres’ plastination process. The non-plastinated bamboo is referred to as virgin bamboo hereafter. As mentioned in Section 1.3, the first step of the S-10 standard procedure involves degreasing to remove lipids. However, in the present case, natural fibres do not possess much lipids so this step was skipped, and the process started with the second step, i.e., acetone dehydration. Details of the experimental procedures can be found in Section 3.

### 2.1. Effect of Plastination on Material Physical Parameters

**Figure 1:**
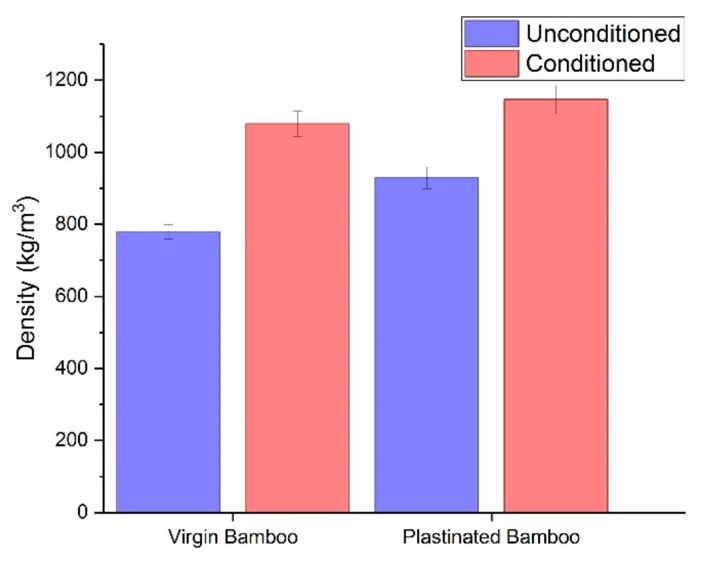
Density of virgin and plastinated bamboo, before and after moisture conditioning.

Figure 1 showed that plastinated bamboo has about 20% higher density than virgin bamboo. This would be due to the excessive silicone that flows into the bamboo fibers and substitutes the water. We can also observe that both virgin and plastinated bamboo have a density increase post-conditioning. This increase in density can be attributed to water absorption, owing to the hydrophilic nature of bamboo. A lesser increase in the density of plastinated bamboo in comparison to virgin bamboo denotes the reduced moisture uptaking capability of plastinated bamboo. However, this also signifies water occupying the internal voids of the cellular structure of plastinated bamboo, besides the presence of silicone at those sites. This discrepancy may be hypothetically attributed to the high water vapour permeability of silicone, which led water molecules to reach the bamboo cell wall and deteriorate it.

**Figure 2:**
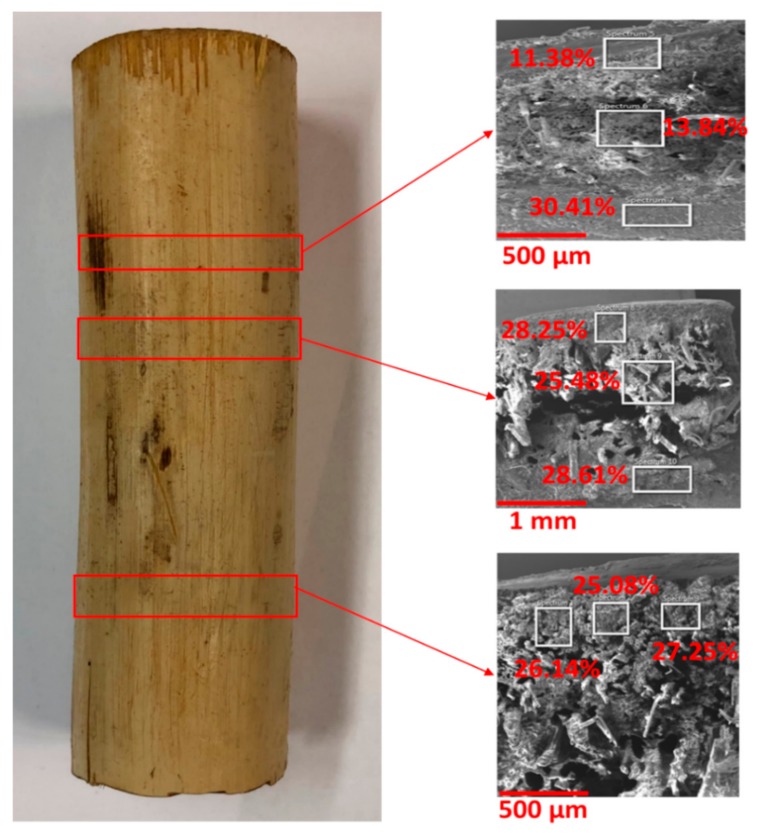
EDS analysis of plastinated bamboo denoting Si content. The variation of silicon denotes a concentration gradient with a lower concentration in the middle of the cross-section.

Table 1 shows that, on average, about 23.94% Si is present in a cross-section of plastinated bamboo vs. 7.5% moisture pre-present before impregnation. It is also worth mentioning that, from the trend seen in the EDS analysis in Figure 2, the Si content in the middle of the cross-section was usually lower than the content on the outer regions. It can thus be suggested that silicone solution may not be able to impregnate completely the innermost regions of the bamboo cross-section using the present S-10 technique.

**Figure 3:**
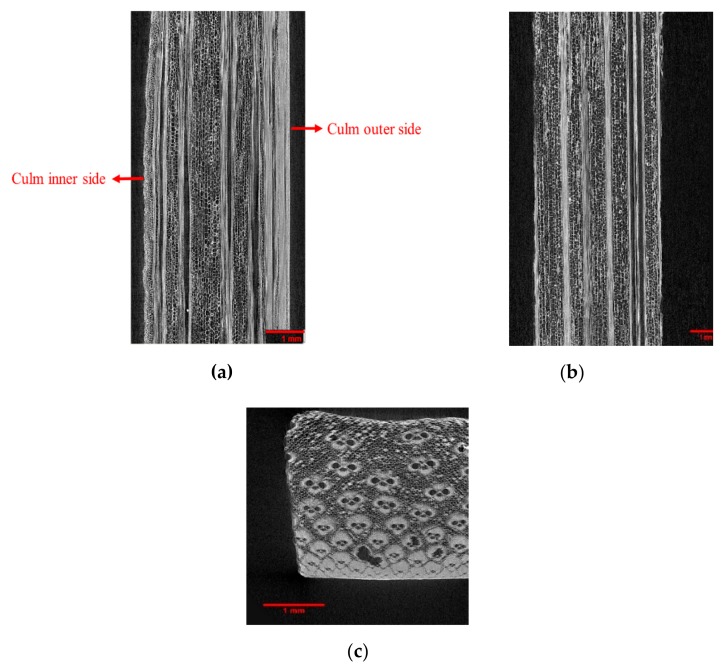
X-ray micro-CT images of virgin bamboo. (**a**) front view, (**b**) side view, and (**c**) top view. The white vertical bundles denote the Xylem while the cellular voids around denote Phloem.

**Figure 4:**
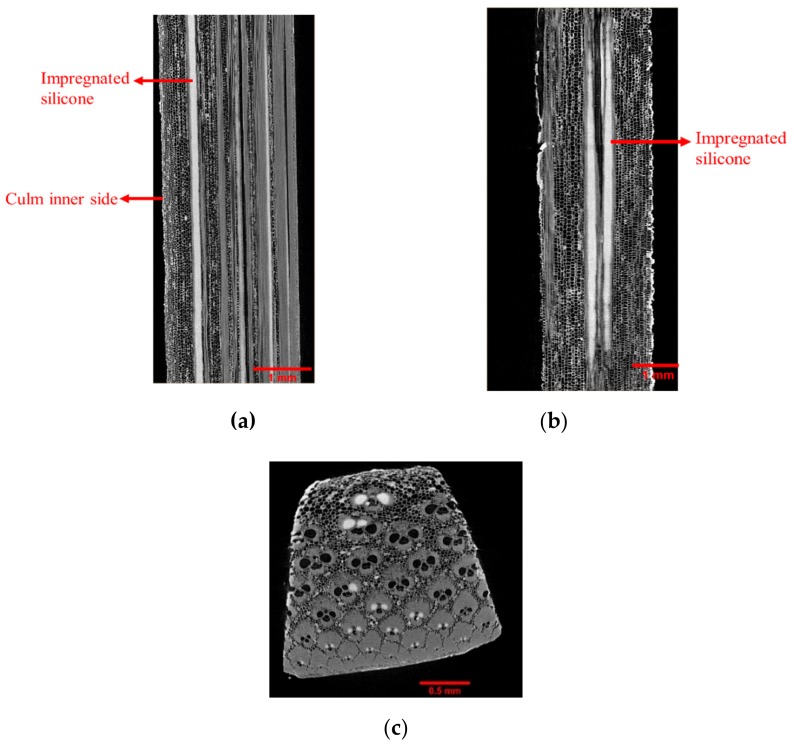
X-ray micro-CT images of plastinated bamboo. (**a**) front view, (**b**) side view, and (**c**) top view. Bright white contrast in vertical bundles denotes an impregnated silicone mixture.

The micro-CT results could well distinguish the long fibre sap (Xylem) from the soft cellular tissues (Phloem) in bamboo, as shown in Figure 3. The higher density of vascular bundles on the outer side of the culm is evident through the higher contract on one of the edges, as highlighted in Figure 3a and Figure 4a. The bright white contrast in the plastinated bamboo micro-CT images (Figure 4) inside the xylem tissue denotes an impregnated silicone mixture. This relates well to the idea of liquid uptake in bamboo since the xylem is responsible for the transport of water across the plant vertically. However, the non-uniform presence of these white spots in the xylem signifies improper impregnation. This also relates to the gradient observed in the concentration of impregnated silicone through the cross-section of plastinated bamboo during the EDS analysis. Comparing Figure 3 and Figure 4, it is also interesting to note that dehydration (moisture removal) has assisted to obtain higher resolution (non-fuzzy) images.

### 2.2. Effect of Plastination on Moisture Behaviour 

**Figure 5:**
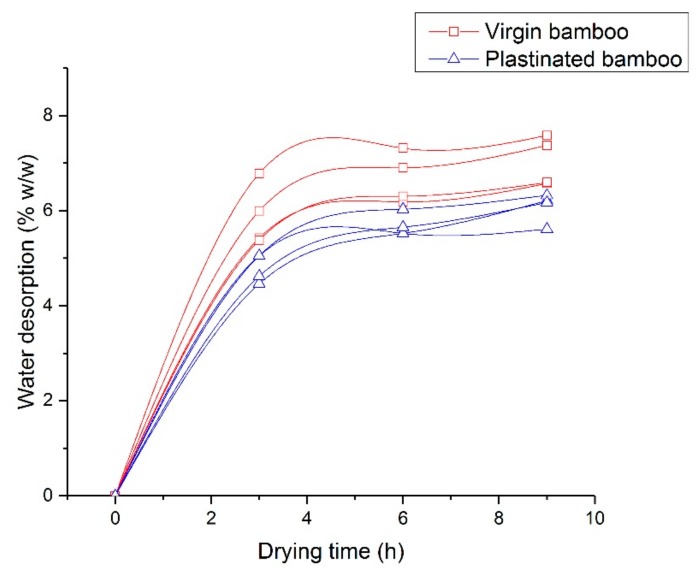
Moisture desorption curves for virgin and plastinated bamboo. Notice the lower residual moisture in plastinated bamboo with reduced moisture diffusivity constant as compared to virgin bamboo.

As previously mentioned, plastinated samples should ideally not have any residual moisture content. Contrary to expectations, plastinated bamboo samples showed some moisture levels that were however lower than what was observed in the virgin bamboo, per Figure 5. It was also apparent from this figure that the slope of water desorption, i.e., the rate of moisture diffusivity constant is lower for plastinated bamboo. On average, plastinated bamboo has relatively about 13% lower moisture content than virgin bamboo with a lower moisture diffusivity constant, denoted by the slope of water desorption during the initial three hours of testing.

**Figure 6:**
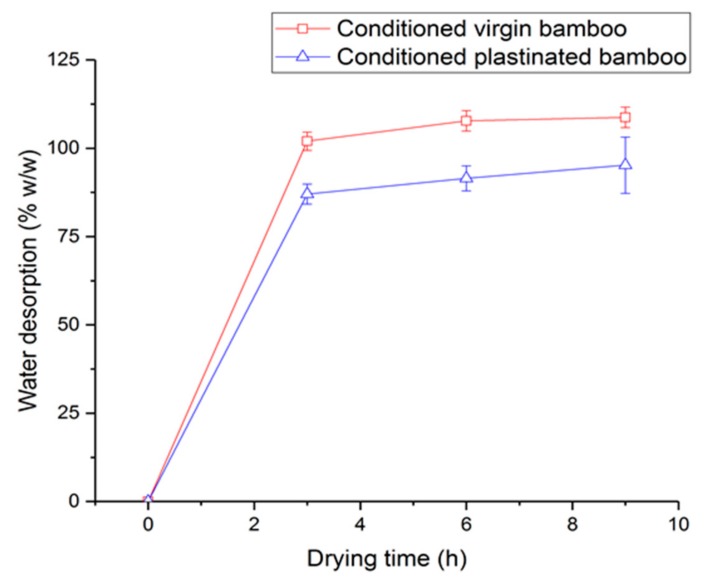
Weight fraction of water desorbed by conditioned virgin and conditioned plastinated bamboo vs. drying time in the oven, showing lower moisture absorbed by plastinated bamboo.

Figure 6 shows the experimental results on the moisture content of conditioned plastinated and conditioned virgin bamboo, with an average moisture content of 109% for a virgin bamboo case. Surprisingly, plastinated bamboo seems to absorb moisture during the water bath with an average moisture content of 95%. It is interesting that the difference between the latter two values (i.e., ~14%) is well within the earlier relative difference seen from unconditioned samples, pointing to the imperfect (local) silicone impregnation as a source, as was visualized through the micro-CT images. This condition may have led to an increase in the overall bound (trapped) water and/or increased transport of free water in bamboo cells during conditioning (note that the water absorption of silicone itself is known to be very low, about 1%) [18]. Further, a smaller desorption slope for plastinated bamboo denotes a lower permeability rate of water absorbed and trapped within the cellular bamboo and silicone. A closer inspection of this figure also reveals that the moisture desorption of plastinated bamboo may be a two-step process where more than half of the water is lost within the first three hours, followed by a gradual loss until equilibrium.

### 2.3. Effect of Plastination on Tensile Properties

As can be seen in Table 2, moisture conditioning led to a 51.3% and 50.7% loss in the tensile strength of virgin and plastinated bamboo, respectively. These changes denote that both plastinated and virgin bamboo are affected by moisture conditioning. Such a reduction in the strength of plastinated bamboo could be attributed to water transport and degrading of the bamboo cell wall, as water acts as a plasticizer to cellulose. However, Table 2 also shows that plastination not only increases the tensile strength of unconditioned bamboo, but even after moisture conditioning, the tensile strength of plastinated bamboo is still 73% higher than that of virgin bamboo. Thus, it can be concluded that, although moisture conditioning decreases the tensile strength of plastinated bamboo, plastinated bamboo still seems to be a much more durable option overall as it can support greater loads under moisture. 

**Figure 7:**
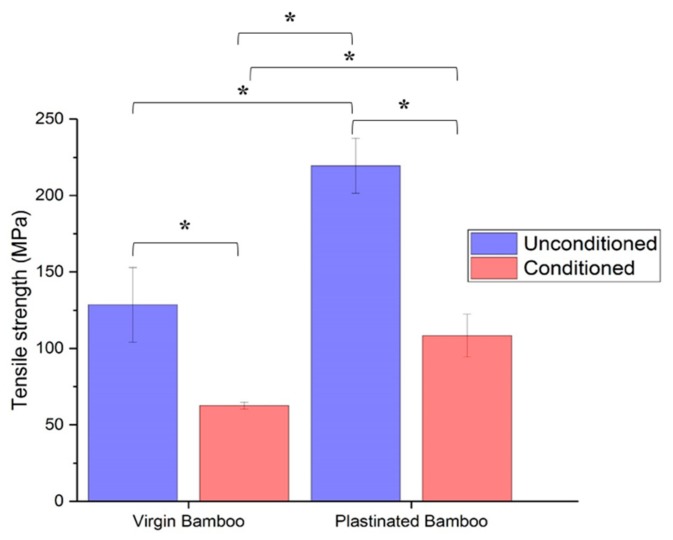
Tensile strength of virgin and plastinated bamboo, before and after moisture conditioning. * indicates a significance level with *p*-value < 0.05.

Further, the results from two-way ANOVA in Figure 7 suggest that both of the study factors along with their interaction are statistically significant at a 5% significance level. Plastination significantly increases the strength of bamboo, and moisture conditioning significantly decreases the strength of plastinated and virgin bamboo. Interestingly, the interaction of these factors suggests that the strength of moist virgin bamboo was significantly lower than dry plastinated bamboo, which could explain the plasticization of cellulose in moist bamboo and the absence of silicone present to uniformly distribute the load. Therefore, following plastination, a significant increase in the tensile strength and moisture durability of bamboo can be observed.

Although the decrease in the tensile strength of conditioned virgin and plastinated bamboo is similar, a cross-comparison study from Table 2 illustrates that the final strength of conditioned plastinated bamboo is about 82% of the strength of virgin unconditioned bamboo. Meanwhile, the tensile strength of virgin conditioned bamboo is only 28.4% of the tensile strength of unconditioned plastinated bamboo. Thus, even after moisture conditioning at elevated temperature, plastinated bamboo retains 82% of the tensile strength of original unconditioned bamboo.

**Figure 8:**
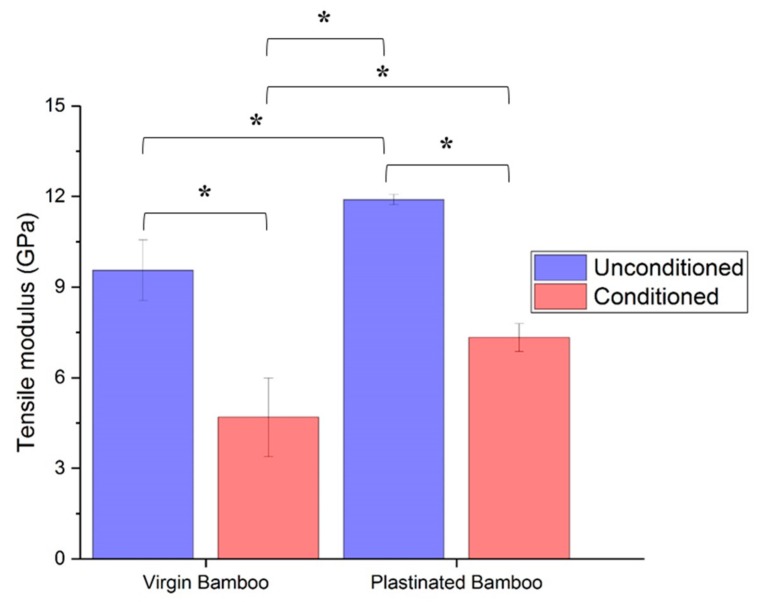
Tensile modulus of virgin and plastinated bamboo before and after moisture conditioning. * indicates a significance level with *p*-value < 0.05.

In terms of modulus, however, the fall in stiffness of conditioned plastinated bamboo is much lower at 38%, as compared to 51% for virgin bamboo as shown in Table 3, as a result of plasticization. Moreover, the observation that conditioned plastinated bamboo provides a 55% higher modulus than conditioned virgin bamboo definitely deems plastination suitable for bamboo processing to enhance its strength and modulus in both moisture conditioned and unconditioned environments. The results obtained for a two-way ANOVA on the tensile modulus datasets were very similar to what was observed for tensile strength, as shown in Figure 8. Thus, it can be seen that moisture conditioning significantly affects the tensile modulus of plastinated and virgin bamboo and that the modulus of moist plastinated bamboo is significantly different from moist virgin bamboo at the 5% significance level.

Further, a cross-comparison of modulus from Table 3 reveals that moistened plastinated bamboo has about 23% lower modulus than unconditioned virgin bamboo, while this percentage is 60% in the case of virgin conditioned and unconditioned plastinated bamboo. Conditioned plastinated bamboo retains 77% of the stiffness of original bamboo as opposed to only 49% for conditioned virgin bamboo.

The fall in the strength and modulus of bamboo after conditioning may be explained by the fact that water presence dramatically softens the cell walls. During the plasticization, cellulose present in bamboo is affected by water, and the hydrogen bonds between different polymer chains in cellulose can break. Subsequently, hydrogen bonds form with water instead, as they are small, polar molecules and hence can penetrate into the polymer chains. These water molecules form hydrogen bonds with cellulose, stronger than those existing between cellulose and cellulose. This softens the cellulose micro-fibrils as they are no longer so strongly bonded to each other, which results in the decreased stiffness of bamboo. Further, as the water expands the cell wall, there are also fewer cellulose micro-fibrils per unit area. Hence, the strength of the bamboo decreases [19].

## 3. Methods

### 3.1. Plastination Procedure

#### 3.1.1. Acetone Dehydration

The idea behind acetone dehydration is to diffuse acetone into the cells to replace the existing water. To this end, the raw bamboo culms and fibre bundles were completely immersed in a 100% concentrated solution of acetone. The acetone container was placed in a deep freezer to maintain a temperature of −25 °C to prevent the evaporation of volatile acetone. The acetone in the container was stirred once daily and the first acetone concentration check was performed after three days of bamboo immersion. Unlike the bulky and complex human body parts that contain much higher aqueous content, the acetone concentration in the container after three days was greater than 99%. After ensuring the acetone concentration was greater than 99%, the bamboo samples were taken towards the next crucial step of plastination, i.e., silicone impregnation.

#### 3.1.2. Forced Impregnation

**Figure 9:**
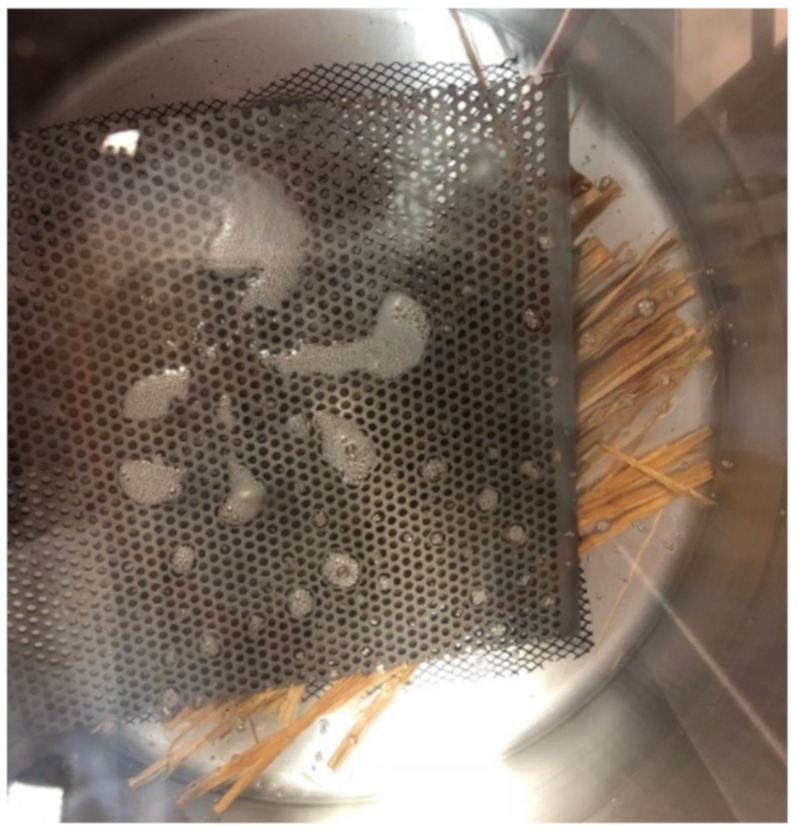
Bubble formation in the silicone chamber due to vacuum pressure reduction.

A room temperature impregnation process was followed in this study in which a high boiling point and low vapour pressure mixture was impregnated into the cells of the specimen by vaporizing the low boiling point and high vapour pressure acetone. To achieve this impregnation level, the dehydrated samples from the acetone bath were quickly immersed into the silicone mixture. These samples were kept immersed in the silicon mixture overnight to achieve an equilibrium between the concentrations of the acetone and silicone mixture. On the second day, the pressure chamber was sealed and pressure reduction inside the chamber was performed using a vacuum pump through a catch pot. Initially, the pressure was decreased to 0.045 m Hg and bubble formation was observed as the pressure decreased, as shown in Figure 9. The mixture was then allowed to withstand this pressure until no bubbles were observed on the surface.

The vacuum was then further increased in a similar manner in increments of 0.102 m. The disappearance of bubbles assured that acetone has been removed within the bamboo and has been replaced by silicone. At around 0.711 m (close to perfect vacuum) the disappearance of bubbles was considered as the point of complete impregnation.

#### 3.1.3. Curing

**Figure 10:**
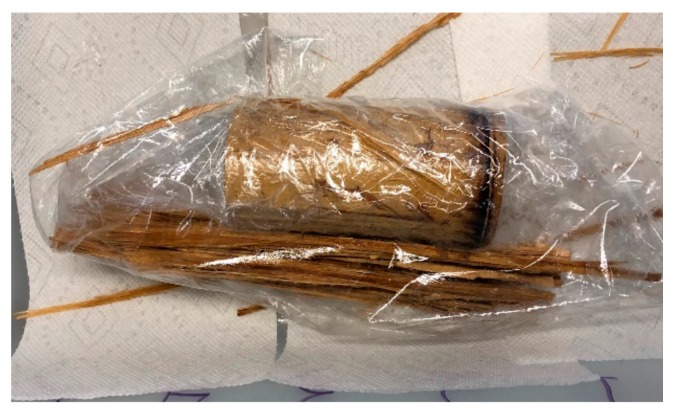
S-3 sprayed bamboo samples covered in a plastic wrap so that the S-3 stays in contact with the impregnated bamboo.

After forced impregnation, the curing of the samples was conducted using a spraying technique. The curing mixture was sprayed on the impregnated samples which initiated a cross-linking reaction between the S-3, S-10, and S-6 mixtures. The samples were later wrapped in a plastic wrap to ensure that S-3 was sealed and stayed in contact with the impregnated bamboo specimens, as shown in Figure 10.

### 3.2. Testing Physical Parameters

The density, microstructure, and chemical composition of the plastinated bamboo were characterized to evaluate the effect of plastination on the physical parameters of bamboo. Micro-tomography was used to visualize the internal structure of plastinated bamboo and to study the extent and interaction of impregnated silicone with the bamboo cellular structure. The density of the unconditioned (dry) and moistened (conditioned, water immersed) plastinated and virgin bamboo samples was calculated to understand how plastination affected the moisture absorption (mass change) and swelling (volume change) in bamboo fibres. To the best of our knowledge, no prior studies investigated the content of silicone that flows into the plastinated sample. Energy dispersive x-ray spectroscopy (EDS) (Oxford Instruments NanoAnalysis, Concord, MA, USA) was conducted on different cross-sections of a plastinated bamboo culm to calculate the amount of silicone flowing into the bamboo. The cross-sections of the bamboo samples were coated with a platinum layer to enable conduction of electrical current [22]. The platinum sputtering thickness was about 6e-6 to 8e-6 m. A Tescan Mira 3 XMU Scanning Electron Microscope was used for the visual investigations. Also for X-ray micro-CT, a Zeiss/Xradia X-400 (ZEISS Xradia 400, Concord, CA, USA), was used. The X-ray source voltage was set to 40 kV with a current of 250 µA. All the micro-CT slices have the spatial resolution of 3.172 µm. No optical filter was used during the scanning process and the 360-degree rotation scan had a step size of 0.144 degrees. Reconstruction software XMReconstructor (V. 8.1, Zeiss/Xradia, Concord, CA, USA) was used to obtain a set of equally spaced 2D images. The resulting slices were then reconstructed into a 3D tomographic image of the sample.

### 3.3. Moisture Conditioning

Six plastinated and virgin bamboo specimens were submerged in tap-water at 20 ± 2 °C (room temperature) for 4 days. The duration, temperature, and other test conditions were determined such that these parameters were consistent with the other ongoing tests as part of a larger study not described herein. The conditioned samples were tested for density change, moisture, and tensile strength. For all these tests, the samples were taken out of the water container and wiped off the excess water from the surface with a dry cloth [23]. These were then quickly tested per the required test procedures. The determination of the moisture content of the wetted samples was performed in accordance with ASTM D4442 (ASTM International, West Conshohocken, PA 19428, USA). This moisture content value provided insight regarding the nature of hydrophilic behaviour of plastinated samples as compared to non-plastinated (virgin) samples and their moisture desorption behaviour.

### 3.4. Tensile Testing

**Figure 11:**
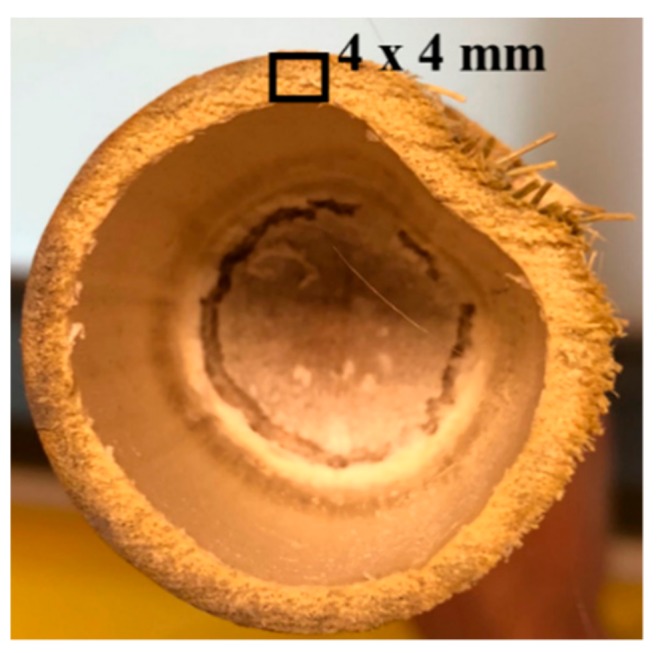
Square cross-section strips extracted from the bamboo culm.

Due to the lack of test codes for the tensile testing of bamboo, a customized sample geometry was developed, with the remaining test guidelines derived from ASTM D4761 (ASTM International, West Conshohocken, PA 19428, USA). This involved 4 × 4-mm sections of bamboo being extracted from the bamboo culm, with height equal to the maximum length of culm, about 120 mm (mostly uniform in all samples), as shown in Figure 11. The tensile strength parameters were then derived using the load-displacement curve generated during the tests. For this test, six replicates for each of the plastinated and virgin bamboo strips were tested.

The analysis of variance (ANOVA) was used for hypothesis testing to determine the effect of various factors and their levels on the tensile properties. For these experiments, a parametric two-way ANOVA was conducted on the tensile strength and modulus properties, with the bamboo type (plastinated vs. virgin) and conditioning type (dry vs. moist) being two factors, each with two discrete levels. As a part of the post-hoc analysis, Tukey’s test was carried out on the statistically significant factors to compare and analyze various mean effects.

## 4. Conclusions

This study showed that the S-10 plastination technique can be applied to bamboo fibers. The tensile tests conducted here denoted that plastination clearly increases the strength and stiffness of bamboo. The moisture tests demonstrated that plastination leads to the enhanced durability of bamboo under moisture conditioning and decreases its hydrophilic tendency to some extent. The absorption of moisture by plastinated specimens and their strength reduction post-conditioning suggest an important issue for investigation in future work. In general, the extent of decay of bamboo (while determining its durability) depends upon the moisture levels in the material and on the microbial environment around it. Lower moisture levels should lead to lower microbial degradation. There is also an abundant potential for the optimization of the plastination process and its application to other natural fibres.

In addition, from an imminent application perspective, the outcome of such research can contribute to the global efforts for the design of future construction projects in response to climate change, by enhancing the resilience of green/semi-green (here due to the replacement of water with silicon) materials against increased humidity and water damage incidents. Mutually, construction is known to be among the key factors that contribute to climate change, due to it being a principal engine for resource extraction and usage. The application of green and semi-green materials can reduce the detrimental, ecological footprints of construction. Finally, despite the reduced water content of fibers, the plastinated materials would be more resilient against microbial degradation, and the actual bio-performance of such new materials can be assessed biologically against toxin production and potential health hazards, especially under natural and extreme weathering cases. Such an interdisciplinary approach can ultimately produce a ‘holistic view’ of the emerging green materials’ life and cost cycles along with their health-related characteristics.

## 5. Patents

The resulting work (plastinated natural fibres) and the application of the plastination technique have been filed as a patent under Canadian patent number PCT/CA2018/051370.

## Figures and Tables

**Table 1:** Chemical composition of plastinated bamboo-derived using EDS.

Statistics	C	O	Si
Max, %	49.51	38.30	30.41
Min, %	36.03	23.06	11.38
Average, %	39.35	33.24	23.94
Standard Deviation, %	4.13	4.32	6.35

**Table 2:** Tensile strength comparison of different bamboo specimens tested.

	Virgin Bamboo (MPa)	Plastinated Bamboo (MPa)	% Relative Change
Unconditioned Bamboo	128.5 ± 24.5	219.5 ± 18	70.8% (increase)
Conditioned Bamboo	62.5 ± 2.21	108.3 ± 14	73.3% (increase)
% Relative Change	51.3% (decrease)	50.7% (decrease)	

**Table 3:** Tensile stiffness comparison of different bamboo specimens tested.

	Virgin Bamboo (GPa)	Plastinated Bamboo (GPa)	% Relative Change
Unconditioned Bamboo	9.6 ± 1.01	11.8 ± 0.17	22.9% (increase)
Conditioned Bamboo	4.7 ± 1.3	7.3 ± 0.46	55.3% (increase)
% Relative Change	51% (decrease)	38.1% (decrease)	
